# In vitro antibacterial effect of probiotics against Carbapenamase-producing multidrug-resistant *Klebsiella pneumoniae* clinical isolates, Cairo, Egypt

**DOI:** 10.1186/s42506-022-00114-4

**Published:** 2022-10-10

**Authors:** Mona Mohiedden Abdelhalim, Ghada Samy Saafan, Hoda Samir El-Sayed, Doaa Mohammad Ghaith

**Affiliations:** 1 Department of Clinical and Chemical Pathology, Faculty of Medicine, Cairo University, Al-Saray Street, Al-Manial, Cairo, 11559 Egypt; 2 Department of Dairy Science, Food Industries and Nutrition Division, National Research Centre, Dokki, Giza, Egypt

**Keywords:** Probiotics, MDR-*Klebsiella pneumoniae*, *Lactobacillus* spp., OXA-48

## Abstract

**Background:**

Searching for a non-antibiotic therapeutic option such as probiotics is gaining momentum nowadays. We aimed to evaluate the in vitro antibacterial ability of cell-free supernatant (CFS) of selected *Lactobacillus* strains (with probiotic properties) against clinical isolates of OXA-48-producing multidrug-resistant (MDR) *Klebsiella pneumoniae* separately and in combination with cefoperazone antibiotic.

**Methods:**

Over a period of 8 months, a cross-sectional experimental study involving 590 *Klebsiella pneumoniae* isolates was done. Our study took place at The Specialized Pediatric Teaching Hospital of Cairo University. Of the 590 *Klebsiella pneumoniae* isolates collected from blood cultures, pus, endotracheal aspirates, and pleural fluid, only 50 unrepeated clinical isolates of MDR *Klebsiella pneumoniae-*producing OXA-48-like detected by CHROMID® OXA-48 (bioMérieux, France) were selected for our study. After determining the minimal inhibitory concentration of CFS of ten *Lactobacillus* strains and cefoperazone each, the synergistic effect of both was tested.

**Results:**

Among ten tested *Lactobacillus* spp., a significant increase in the mean value of inhibition zone diameter with CFS of *L. helveticus* (14.32 mm) and *L. rhamnosus* (13.3 mm) was detected separately. On the contrary, an antagonistic activity against all tested isolates was detected upon combination of *Lactobacilli* with cefoperazone (512 μg/ml). The mean value of inhibition zone diameter of *L. helveticus* CFS+ cefoperazone was (11.0 mm) and for *L. rhamnosus* CFS+ cefoperazone was (10.88 mm) (*p* value <0.001).

**Conclusion:**

The antimicrobial efficiency of using CFS of *Lactobacillus* species separately indicates that these therapies may be a substitute treatment strategy against MDR *Klebsiella pneumoniae*.

## Introduction

Multidrug-resistant (MDR) *Klebsiella pneumoniae* stands out as an “urgent threat to human health,” especially the carbapenamase-producing isolates. Pediatric patients are more vulnerable to complications caused by last resort antibiotics such as colistin [1, 2].

In the trial to find non-antibiotic therapeutic options, probiotic therapy is gaining momentum nowadays. Probiotics are non-pathogenic living microorganisms utilized by different mammalian species they are considered as modifiers of the biological responses. One of the probiotic species of great interest in the food industry are *Lactobacilli* which are facultative anaerobic or microaerophilic, rod-shaped, non-spore-forming bacteria [3–5].

Combined therapy of probiotics and antibiotics has several benefits including treatment of mixed infections, shortening of therapeutic duration, hence preventing emergence of mutant MDR bacteria [6]. The present study was conducted to evaluate the in vitro antibacterial ability of CFS of selected *Lactobacillus* strains (with probiotic properties) against clinical isolates of OXA48-producing MDR *Klebsiella pneumoniae* separately and in combination with cefoperazone antibiotic.

## Methods

### Study design and settings

Out of 590 *Klebsiella pneumoniae* isolated from various clinical samples as a part of the routine hospital laboratory procedure (blood culture, pus, endotracheal aspirates and pleural fluid) over a period of 8 months from February 2017 to September 2017, only 50 unrepeated clinical isolates of MDR-*Klebsiella pneumoniae* were OXA 48 like producers and were selected for our cross-sectional experimental study. All samples were processed in the microbiology laboratory of the Specialized Pediatric Teaching Hospital, Cairo University. The study was approved by the ethical committee of the Clinical and Chemical Pathology Department and patients’ parents provided written informed consent.

### Bacterial isolates

All clinical samples were cultured on blood chocolate and MacConkey agars (Oxoid, England). Identification of all isolates was done by Vitek 2 compact system. Antimicrobial susceptibility testing was done by Kirby-Bauer method where an inoculum density equivalent to 0.5 McFarland was inoculated on Mueller-Hinton agar plates (Oxoid cop. England). Antimicrobial agents used were imipenem 10 μg, meropenem 10 μg, cefotaxime 30 μg, ciprofloxacin 5 μg, trimethoprim sulfamethoxazole 25μg, polymyxin 50μg, piperacillin-tazobactam 85μg, amikacin 30μg, levofloxacin 5μg, cefoperazone75 μg, ceftazidime 30μg, gentamicin 10 μg, ampicillin-sulbactam 20 μg, and amoxacillin-clavulunate 30 μg. Antimicrobial discs were obtained from Oxoid Limited Basingstoke, Hamsphire, England. Antimicrobials were supplied and stored according to the manufacturer’s instructions. *Escherichia coli* ATCC 25922 and *Pseudomonas aeruginosa* ATCC 27853 were used as reference strains for susceptibility testing. The diameters of the inhibition zones were measured for each antibiotic, and MDR *Klebsiella pneumoniae* was identified if the isolate showed acquisition of non-susceptibility to at least one antibiotic of ≥3 different categories [7]. Interpretation of disc diffusion zone diameters was done according to the clinical laboratory slandered institute (CLSI 2017) [8].

#### Detection of OXA-48-like carbapenamase-producing isolates

All *Klebsiella* isolates were directly inoculated on CHROMID® OXA-48 (bioMérieux, France) according to the manufacturers’ recommendations.

#### Determination of minimum inhibitory concentrations (MICs) of cefoperazone

The minimum inhibitory concentrations (MICs) of cefoperazone (Sigma Aldrich, UK) against indicator pathogens were determined by broth micro-dilution method according to *BSAC 2012, CLSI 2017* [8, 9]. The preparation of stock solutions of antibiotic used was conducted using the following formula to determine the amount of powder needed for a standard stock solution:

$$\mathrm{Weight}\ \left(\mathrm{mg}\right)=\mathrm{volume}\ \left(\mathrm{mL}\right)\times \mathrm{concentration}\ \left(\upmu \mathrm{g}/\mathrm{mL}\right)$$

$$\mathrm{Potency}\ \left(\upmu \mathrm{g}/\mathrm{mg}\right)$$

#### Processing of Lactobacilli strains

*Lactobacilli* strains were provided by the National Research Center from different origins as follows:

1. The Northern Regional Research Laboratory “NRRL” Illinois, USA: *Lactobacillus reuteri* B-14171, *Lactobacillus salivaricus* NBIMCC-1589, *Lactobacillus brevis* NBIMCC-3448, *Lactobacillus carvutus* NBIMCC4562, *Lactobacillus gasseri* NBIMCC-1589, *Lactobacillus rhamnosus* B-445, and *Lactobacillus helveticus* CNRZ-32
2. *Lactobacillus acidophilus* was obtained from Chr. Hansen’s Lab., Denmark
3. *Lactobacillus plantarum* and *Lactobacillus casei* were provided by the Faculty of Agriculture Ain-Shams University.

Cell-free supernatant was prepared using Man-Rogosa-Sharpe broth (Sigma Aldrich) according to De Man et al., Liasi et al., and Eid et al. [10–12].

#### Detection of in vitro antibacterial activity of Lactobacillus spp. by agar-well diffusion method

Screening of antimicrobial activity of different *Lactobacillus* spps. against 10 isolates of MDR *Klebsiella pneumoniae-*producing OXA-48 (selection was done upon the highest MIC values to B lactams) to determine which types of CFS were the most potent to be used in combination with cefoperazone to evaluate the synergistic action.

The agar-well diffusion method was used for antimicrobial testing. The selected isolates of MDR *Klebsiella pneumoniae* were adjusted to 0.5 MacFarland suspension in sterile tubes. The isolates were swabbed individually on the surface of sterile Muller Hinton agar plates using a sterile cotton swab. Agar wells were prepared with sterilized cork-borer with a 10-mm diameter. One hundred microliters of CFS formulated in each of *Lactobacilli* spp. was added to the agar wells in the plate which were incubated at 37 °C for 24 h. The presence of inhibition zone was considered as the indicator of antimicrobial action that is reported in millimeters, and the zone diameter obtained was measured and interpreted following Shokryazdan et al., Mogna et al., Prabhurajeshwar et al., and Abed et al. [13–16].

Quality control strain *Klebsiella pneumoniae* (ATCC 35657) was used for comparison with CFS of different *Lactobacillus* strains.

#### Antimicrobial effect by using CFS of the most potent Lactobacillus strains (L. helviticus and L. rhamnosus alone and in combination with cefoperazone

Six wells were punched with sterile cork borer in Muller Hinton agar, after seeding the plates with fresh culture organism adjusted to 0.5 MacFarland suspension in sterile tubes. Wells were filled with 100μl CFS of probiotic *Lactobacillus helviticus*, 100μl CFS of probiotic *Lactobacillus rhamnosus*, 100μl of MIC= 512 μg/ml of cefoperazone), 50μL CFS of probiotic *Lactobacillus helviticus*+, 50μL of MIC= 512 μg/ml of cefoperazone, 50μL CFS of probiotic *Lactobacillus rhamnosus*+, 50μL of MIC= 512 μg/ml of cefoperazone, and 100μl of distilled water as a negative control. Then, the plates were covered and incubated at 37°C for 18 h. The inhibition zone around the well was measured in millimeter, and all isolates were done in duplicates.

### Statistical analysis

Data management and statistical analysis were performed using Statistical Package for Social Sciences (SPSS) vs. 23. Numerical data were summarized using means and standard deviations. Categorical data were summarized as numbers and percentages. The zone of inhibition was compared between different groups using Kruskal-Wallis test. Categorical variables were compared using chi-square test. All *p* values are two-sided. *P* values < 0.05 were considered significant.

## Results

**Fig. 1:**
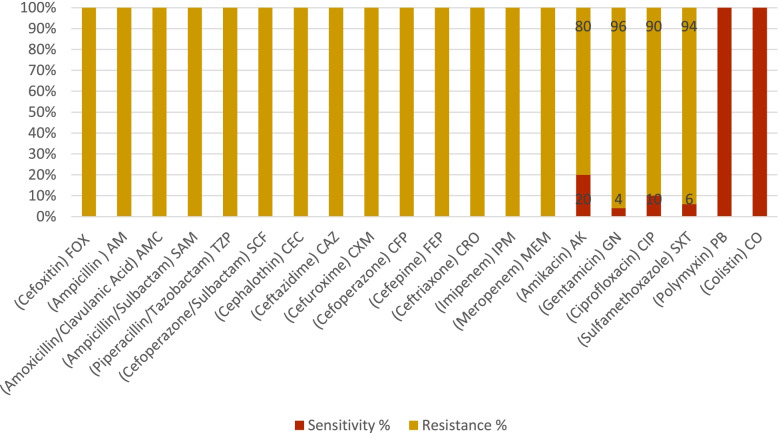
Antibiogram profile of all *Klebsiella pneumoniae* tested isolates

*Klebsiella pneumoniae* clinical isolates were isolated from blood cultures (18/50) of 36%, endotracheal aspirates of 32% (16/50), wound of 20% (10/50), pleural fluid of 6% (3/50), and pus samples of 6% (3/50). The antibiogram of the tested isolates is shown in Fig. 1.

All children were admitted in the intensive care unit (ICU) (8 died during the study) and had high C-reactive protein levels, while 86% (43/50) of cases had shift to left findings in their complete blood pictures. Indwelling devices were the most significant risk factor used for 98.0% (49/50) of patients in the form of urinary catheter—central venous catheter—endotracheal tubes, followed by operative interventions in (38/50) of cases.

Among all tested *Lactobacilli*, the CFS of *Lactobacillus rhamnosus B-445 and Lactobacillus helveticus CNRZ-32* showed the most accepted growth inhibitory effect against MDR isolates as shown in Table 1.

**Table 1.** Screening of the inhibition zone of growth in (mm) for isolated MDR *Klebsiella pneumoniae* isolates versus standard strain *Klebsiella pneumoniae* ATCC 35657 by different probiotic *Lactobacilli* spp.

Strains supernatant	***K.*** 1	***K.*** 2	***K.*** 3	***K.*** 4	***K.*** 5	***K.*** 6	***K.*** 7	***K.*** 8	***K.*** 9	***K.*** 10	***K.*** ATCC 35657
*L. acidophilus*	0	0	0	0	0	0	0	0	0	0	0
*L. casei*	10mm	0	0	0	9mm	0	0	0	9mm	0	0
*L.rhamnosus*	12mm	10mm	14mm	12mm	10mm	12mm	12mm	11mm	13mm	13mm	12mm
*L.reuteri*	0	0	0	10mm	0	0	0	10mm	9mm	0	0
*L. plantarum*	0	0	9mm	9mm	0	0	0	0	0	0	0
*L. salivaricus*	0	0	0	0	0	0	0	0	0	0	0
*L. brevis*	0	0	0	0	0	0	0	0	0	0	0
*L. carvutus*	10mm	10mm	9mm	0	0	0	0	0	0	0	0
*L. gasseri*	0	0	0	0	0	0	9mm	9mm	0	0	0
*L. helveticus*	14mm	12mm	13mm	13mm	12mm	13mm	12mm	13mm	14mm	14mm	13mm
Combined cefoperazone and CFS	0	0	0	8mm	0	0	0	0	9mm	0	0

*K Klebsiella pneumoniae*, *L Lactobacillus*

**Fig. 2:**
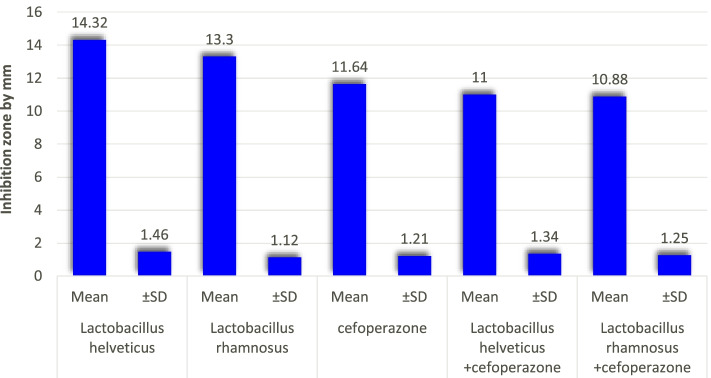
Antimicrobial effect of *Lactobacillus helveticu*s and *Lactobacillus rhamnosus* versus the minimal inhibitory concentration of cefoperazone (512 μg/ml) alone and in combination with each of them

Most of the MDR isolates exhibited high-level resistance by broth microdilution assay shown by visual turbidity. As 76% (38/50) of isolates showed no sensitivity to the highest concentration of antibiotic cefoperazone so (MIC > 512 μg /ml) and 24% (12/50) of isolates became sensitive at the highest concentration of cefoperazone antibiotic (MIC= 512 μg/ml). So, we used the concentration of (MIC= 512 μg/ml) in the combination trials by agar well diffusion method with CFS of (*L. helveticus*) and (*L. rhamnosus*). The results of antimicrobial effect of *Lactobacillus helveticu*s CFS and *Lactobacillus rhamnosus* CFS versus MIC of cefoperazone at (512 μg/ml) alone and in combination is shown in Fig. 2.

## Discussion

Carbapenem resistant *Enterobacteriaceae* is especially problematic in Egypt. As per a recently published study which used the national healthcare associated infection surveillance data from 2011 to 2017 in the Egyptian intensive care units, 54.1% of *Enterobacteriaceae* were carbapenem resistant dominated by *Klebsiella* spp. Moreover, previous studies in our institute showed alarming predominance of OXA-48-producing *Enterobacteriaceae* colonization in intensive care units [1, 2, 17–21].

In our study, the incidence of MDR *K. pneumoniae* out of the total *Enterobacteriaceae* isolates was 73% with 38% (19/50) of cases were MDR isolates and 62% (31/50) of cases were XDR according to the antibiogram in the study period from February to September 2017. Several studies approved the anti-biotherapy based on the antibiogram of the causative agent due to the good correlation between the in vitro and in vivo results [22].

According to the pediatric guidelines of antibiotic choice in our institute, all our patients received the first line of antibiotics including (amoxicillin/clavulonate-ampicillin/sulbactam-amikacin–gentamycin-cefotaxime); meanwhile, 94% (47/50) of patients were shifted to the second line which includes (ciprofloxacin-piperacillin/tazobactam–teicoplanin–vancomycin-imipenem) based on the indicators of infection (fever, high CRP, and shift to left).

Endimiani et al. mentioned the misuse of the commercially available cephalosporins/-β-lactamase inhibitors such as clavulanic acid, sulbactam, or tazobactam limits their clinical application [23]. Yet, cephalosporins are preferred choice in children due to their safety. In our study, cefoperazone was selected based on its welfare in children and availability in our country. All MDR isolates exhibited high-level resistance to the highest concentration of cefoperazone (MIC = 512 μg/ml). Out of 50 isolates, 76% (38) of cases did not show sensitivity to the highest antibiotic concentration (MIC =512 μg/ml).

Combination is one of the competing measures of resistance, e.g., the most common successful combination is polymyxin with either tigecycline or a carbapenem. In our study, the third line antibiotics was used in 74% (37/50) of cases as the last resort of antibiotics, in the form of combination (polymyxin+ meropenem) with success rate 56% in concordance with Zusman et al. study in which the success rate reached 73% when polymyxin was used in combination with carbapenems [24]. Meanwhile, Lee et al. showed that polymyxin monotherapy was associated with higher treatment failure rates in comparison to polymyxin-based combination therapy (29 vs 73%; *p=*0.02) [25].

Another suggested method to fight resistance is the usage of non-antibiotic treatments such as probiotics. Choosing probiotics for our study was in accordance with the Food and Agriculture Organization of the United Nations (FAO) and the World Health Organization (WHO). The present study explored that the active supernatants of the potential isolates *L. helveticus* and *L. rhamonosus* have a good inhibitory effect on the tested pathogenic clinical isolates of *K. pneumoniae* and on the standard quality control *Klebsiella* strains concurrently. The difference in antimicrobial activity of our strains could be due to the production of a wide range of antimicrobial substances, including lactic and acetic acids, ethanol, bacteriocins by homo-fermentative and hetero-fermentative *Lactobacilli*, or by competing with pathogens for nutrients and other growth factors and suppressing the growth of pathogenic bacteria [26].

Using antibiotics’ combinations can be a tool to avoid clinical resistance (inhibition of organism by safe antibiotic concentration), our study on 50 MDR isolates showed that the mean of inhibition zones of *L. helveticus* CFS was (14.32 mm) and *L. rhamnosus* CFS (13.3 mm) with good antimicrobial activity against all MDR isolates compared to mean results of highest concentration of cefoperazone antibiotic (512μg/ml) which was (11.64 mm) with a statistical highly significant difference (*p* value <0.001).

Using CFS and live cells in a study by Fernández et al. [27] showed that the strongest antimicrobial activity was observed when the live cells of *L. rhamnosus* were used compared to other *Lactobacilli* species. This was explained as bacteriocins is produced significantly by culturing on agar medium by a greater amount than that in liquid culture. Also, Al-Mathkhury et al. mentioned that bacteriocin produced by *L. acidophilus* isolated from yogurt was shown to significantly inhibit the growth of clinical isolates of MDR gram-negative bacilli isolated from urinary catheters [27, 28].

In accordance with our study, Perez et al. proved that crude CFS containing bacteriocin from lactic acid bacteria (LAB) will be the next-generation antibiotic, which can be used to target the MDR gram-negative pathogen-producing β-lactamases [29].

In a study by Griffiths et al., the most significant effect demonstrated by *L. helveticus* was due to its ability to produce bioactive compounds during milk fermentation. The industrial importance of *L. helveticus* depends on its effective proteolytic system, which seems to be the most efficient between LAB, with a big impact on the production of bioactive peptides [30]. Another study by *Monteagudo-*Mera et al. showed that probiotic properties of *L. rhamnosus* from pharmaceutical sachets showed growth inhibitory property against gram-positive as well as gram-negative bacterial strains [31].

Pena-Miller et al. mentioned that the preliminary treatment of bacteria with the strongest synergistic drug combinations will kill the vulnerable bacteria leaving the resistant bacteria to grow at a faster rate in days 2 through 5, compared with the population that was initially treated by just a single drug or a weaker synergistic combination preferring selective overturn for resistance and more growth rate to the susceptible strains [32].

### Limitations of the study

Although using probiotics in treating MDR bacteria is promising yet, concerns have been raised regarding the potential pass of resistance genes through horizontal gene transfer between different species. More studies including larger numbers of probiotic strains is needed.

## Conclusion

Although combination between CFS and cefoperazone (512 μg/ml) showed antagonistic activity against all MDR isolates, the antimicrobial efficiency of using CFS of *Lactobacillus* species separately indicates that these therapies may offer the basis to substitute therapeutic strategy against MDR bacteria.

## Data Availability

In this study, all the data and materials are included. If more information is needed, please contact the author for data requests.

## Abbreviations

CFS: Cell-free supernatant
MDR: Multidrug-resistant
CLSI: Clinical laboratory slandered institute
MIC: Minimum inhibitory concentration
ICU: Intensive care unit
WHO: World Health Organization
LAB: Lactic acid bacteria
